# SETD2 and EZH2: Two epigenetic drivers of prostate cancer

**DOI:** 10.7150/jca.115715

**Published:** 2025-07-28

**Authors:** Jiamin Wang, Longquan Xiang, Haiyan Zhang, Xiangyu Zhang

**Affiliations:** 1 School of Clinical Medicine, Shandong Second Medical University, Weifang, Shandong 261053, P.R. China.; 2 Department of Pathology, Jining No.1 People's Hospital, Jining, Shandong 272000, P.R. China.; 3 Zoucheng People's Hospital, Zoucheng, Shandong 273500, P.R. China.

**Keywords:** SETD2, EZH2, Prostate cancer, Inhibitor, Epigenetic modifications.

## Abstract

Prostate cancer is a prevalent malignancy among men, characterized by complex mechanisms underlying metastasis and treatment resistance. Epigenetic modifications play a crucial role in regulating prostate cancer progression, particularly involving histone methyltransferases such as SET-domain containing 2 (SETD2) and Enhancer of Zeste homolog 2 (EZH2). SETD2 contributes to chromatin stability by catalyzing the trimethylation of histone H3 lysine 36 (H3K36me3), and its downregulation is strongly correlated with increased invasiveness and epithelial-mesenchymal transition in prostate cancer. Conversely, EZH2, the catalytic subunit of Polycomb Repressive Complex 2, mediates gene silencing through H3K27me3 modification and is frequently overexpressed in advanced disease, promoting tumor metastasis and resistance to therapy. Notably, SETD2 regulates EZH2 stability through direct protein interactions, highlighting a coordinated epigenetic regulatory axis. Multi-omics studies have revealed that SETD2 loss induces aberrant DNA methylation and activates oncogenic signaling pathways, whereas EZH2 overexpression cooperates with PI3K-AKT pathway dysregulation to drive castration-resistant prostate cancer. Although inhibitors targeting SETD2 (e.g., EZM0414) and EZH2 (e.g., tazemetostat) demonstrate antitumor activity in preclinical models, their clinical efficacy remains constrained by drug resistance and tumor microenvironment heterogeneity. Emerging evidence suggests that combining epigenetic therapies with immunotherapy may enhance therapeutic outcomes. This review comprehensively systematically examines the molecular mechanisms underlying the SETD2/EZH2 axis in prostate cancer, providing a theoretical foundation for developing precision therapies based on SETD2- or EZH2-mediated epigenetic modifications.

## 1. Introduction

Prostate cancer remains one of the most commonly diagnosed malignancies in men and ranks among the leading causes of cancer-related mortality worldwide 1. Among the numerous mechanisms involved in its development and progression, epigenetic regulation has emerged as a significant contributor. The concept of epigenetics was proposed by Conrad Waddington in 1939. Epigenetic modifications refer to heritable changes in gene expression that do not involve alterations in the DNA sequence itself. These changes—such as DNA methylation, histone modification (methylation and acetylation), and chromatin remodeling—play essential roles in development and disease, often preceding genetic mutations in cancer initiation. Epigenetic changes manifest earlier than genetic changes. Studies exploring the mechanisms underlying prostate cancer metastasis have focused on the role of epigenetic regulation and its modifications. Histone methyltransferases (HMTs) are comprised of histone lysine methyltransferases (KMTs) and protein arginine methyltransferases (PRMTs). Both SETD2 and EZH2 are KMTs, and involved in prostate cancer progression.

One of the key players in epigenetic regulation is the histone methyltransferase SET-domain containing 2 (SETD2), the sole enzyme known to catalyze histone H3 trimethylation at lysine 36 (H3K36me3). The SETD2 gene is located on chromosome 3, p21.31 2, and is widely recognized for its tumor-suppressive functions. Aberrant expression of SETD2 has been linked to poor prognosis, chromatin instability, increased tumor aggressiveness, increased metastatic potential, and resistance to therapy across various cancers, including prostate cancer 3-5. In particular, reduced or absent SETD2 expression has been associated with enhanced invasiveness and epithelial-mesenchymal transition (EMT), underscoring its critical role in disease progression 6.

By contrast, Enhancer of Zeste homolog 2 (EZH2), the catalytic subunit of Polycomb Repressive Complex 2 (PRC2), is responsible for catalyzing mono-, di-, and tri-methylation of histone H3 lysine 27 (H3K27me1-3) 7-8. EZH2 is a well-established oncogene whose overexpression leads to transcriptional silencing of tumor suppressor genes, thereby promoting proliferation, metastasis, and resistance to hormonal therapies in prostate cancer 9-11. Studies have uncovered that SETD2 binds to EZH2 and promotes its degradation through lysine 735 (K735) methylation, thereby preventing a shift toward a hyper-repressive H3K27me3 chromatin state 12.

This review aims to elucidate the epigenetic regulation of SETD2 and EZH2 in prostate cancer, focusing on their molecular mechanisms of action, involvement in metastasis, effects on histone modification, and impact on gene expression regulation to promote or inhibit prostate cancer metastasis, to offer a basis for prostate cancer diagnosis and treatment, as well as for the research and development of novel drugs.

## 2. SETD2 and Prostate Cancer

### 2.1 Structure and function of SETD2

**Figure 1:**
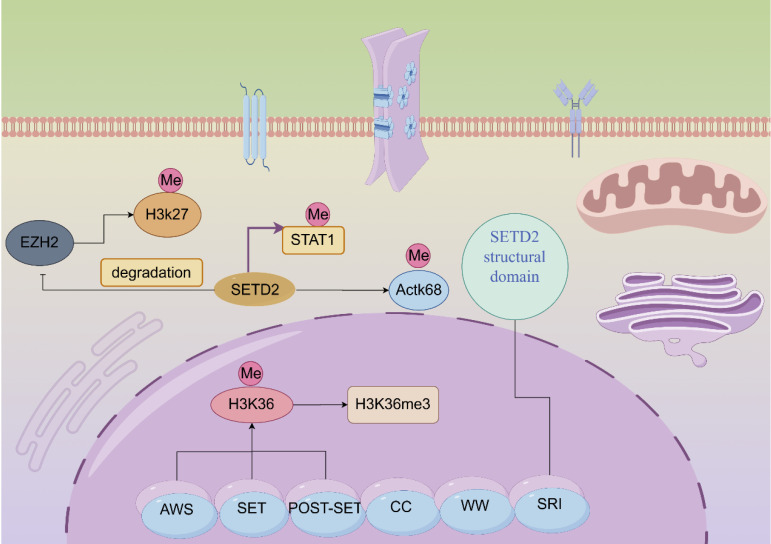
Molecular structure diagram and functional schematic of SET-domain containing 2 (SETD2). Activation is indicated by arrows and suppression by blocking lines.

*SETD2* is located on the human chromosome 3p21.31 region 2. *SETD2* contains multiple exons and introns, a structural feature that enables the generation of multiple transcripts during post-transcriptional processing of genes through variable splicing and other means. These different transcripts may perform diverse functions across cellular environments or under different physiopathological conditions. Human SETD2 protein possesses multiple functional domains that are more conserved, mainly including the AWS (SET-related)-SET-PS (post-SET) structural domain, the WW structural domain, SRI (Set2-Rpb1 interacting structural domain), the SETD2-hnrnp interacting (SHI) structural domain and a large unstructured n-terminal structural domain 13. Different structural domains have their functions and missions. Among them, the n-terminal region of SETD2 plays a decisive role in maintaining the stability of SETD2. The removal and loss of the n-terminal fragment results in compromised stability of SETD2, which significantly decreases the expression of H3K36me3 14. SRI domain of SETD2 is indispensable for SETD2 functions, SRI domain's direct association with phosphorylated Pol II (RNAPII-pCTD) can lead to targeted gene transcription elongation 15. SRI domain contain 15 amino acids, can recognize the linker DNA of chromatin. SRI can control substrate specificity. SRI domain can affect the half-life of SETD2 protein through proteasome-dependent pathway, and influence the catalytic activity of SET domain. And SRI domain affect SETD2's ability to methylate non-histone substrates. A pathogenic mutation (R2510H) in the SRI domain impairs SETD2 ability to methylate α-tubulin at lysine 40 during mitosis 16. Cryptic transcription is the process of transcription occurring at unexpected or non-canonical sites within the genome. SETD2 can regulate cryptic transcription and pre-mRNA splicing to alter target gene functions 17. SETD2-mediated H3K36me3 serve as a binding site for various chromatin regulators that can inhibit cryptic transcription. One study shows that cryptic 5' splice sites are activated when they bind U1 snRNP much stronger than authentic 5' splice sites 18. SETD2 prevents cryptic transcription using a different H3K36me3-mediated mechanism, and is independent of histone deacetylation. This process is closely related to interaction of SETD2 with DNA methyltransferase 3B (DNMT3B). But one study showed that SETD2-mediated H3K36me3 could induce histone deacetylation in higher eukaryote to inhibit cryptic transcription 19. SETD2 mutation often leads to defects in transcript processing, including cryptic transcription. SETD2 interacts with hnRNP L to regulate alternative splicing of pre-mRNA. One study shows that SETD2 knockdown can induce alternative splicing in PKM2, TPM1 and TPM2 genes 20. SETD2 plays an invaluable role in mammalian epigenetic regulation. SETD2 catalyzes histone methylation and interacts with RNA polymerase II to mediate transcriptional elongation. We noted that the development of several tumors is associated with mutations in SETD2; for instance, gastric, kidney, and lung cancers 6. SETD2 is the only histone methyltransferase (HMTase) that catalyzes the trimethylation of lysine 36 on histone H3 (H3K36me3), which results in the epigenetic marking of H3K36me3 21. Among the epigenetic marks, H3K36me3 plays a role in transcription elongation, selective splicing, and DNA repair (**Figure 1**) 21-22. Sometimes, low expression of SETD2 can confer advantages of proliferation, colony formation, migration and invasion for cancer cells.

### 2.2 Epigenetic regulation of SETD2 in prostate cancer and metastatic mechanisms

The majority of SETD2 mutations were heterogenous, and almost half of these mutations were nonsense, frameshift, splice site mutations or deletions, which were loss-of-function alternations. SETD2 mutant patients usually harbored high mutation burden and microsatellite instability. SETD2 mutation occurred in a dispersed manner across the whole sequence. SETD2 mutation of loss-of-function was related to prostate cancer progression 23. Several studies have demonstrated significantly lower levels of SETD2 expression in prostate tissues when compared to that in normal prostate tissues. The downregulation of SETD2 is closely associated with prostate cancer development, which reinforces the idea of SETD2 exerting a biological function as a tumor suppressor. Moreover, decreased SETD2 expression decreases the H3K36me3. In contrast, the SETD2-H3K36me3-signaling axis is closely related to epigenetic regulation, which, in turn, affects chromatin status. In addition, SETD2 loss may be associated with pancreatic cancer because its loss leads to the ectopic expression of H3K27me3 and H3K27ac, which would contribute to the occurrence of immune escape in pancreatic ductal carcinoma 24-25.

DNA methyltransferase 3β (DNMT3B) is a methyltransferase, and SETD2 promotes DNMT3B recruitment 24. SETD2, H3K36me3, DNMT3B, and DNA methylation interact in epigenetic regulation, enabling the fidelity of gene transcription 26. SETD2 deficiency disrupts this balance, resulting in hypermethylation or hypomethylation of histone proteins *in vivo*, which can induce malignant changes as cancer cells gain the ability to invade and migrate.

Moreover, in the absence of the key oncogenes, SETD2-H3K36me3 is similarly absent, which then constructs a possible association between SETD2-H3K36me3 and carcinogenesis 24. There are several important structural domains in the SETD2 protein that act synergistically to enable SETD2 to recognize and bind to the histone H3 tail, which, in turn, catalyzes H3K36me3 27. When SETD2 is deficient, it inhibits the expression of the epithelial marker E-cadherin, while upregulating the expression of mesenchymal markers N-cadherin and Vimentin, which promotes the EMT of prostate cancer cells, so that the cells acquire a stronger migratory and invasive ability and thus become prone to metastasis. SETD2 can maintain a normal epithelial cell phenotype by regulating the histone methylation status of the promoter regions of the related genes. When the SETD2 expression is reduced, it fails to effectively inhibit the expression of mesenchymal-related genes, leading to EMT in prostate cancer cells and thereby promoting tumor metastasis. SETD2 mutation often leads to resistance to DNA-damaging agents, such as doxorubicin, cytarabine, and etoposide. SETD2 loss decreases the activation of the DNA damage response after exposure to cytotoxic agents. One study showed that SETD2 missense mutation (p.T1171K) affect CREB1 phosphorylation and mediate the cisplatin resistance 28. There are also a study showing that SETD2 regulate MSH6 gene expression to mediate temozolomide resistance 29. SETD2 inactive mutation is involved in sunitinib resistance in renal cell carcinoma, may be attributed to reduced signal transduction of MCL-1. SETD2 knockout can inhibit ERK activation induced by cisplatin and upregulate Bcl-xL to induce cisplatin resistance. One study indicates that SETD2 loss in cancer cell can shape cancer-associated fibroblasts heterogeneity to support cancer progression 30. SETD2 inactivation can affect tumor immune microenvironment to enhance neutrophil recruitment. SETD2 may influence the functions of CD8+ T cell, CD4+ T cell and macrophages. SETD2 can suppress the Th17 cell development and promote iTreg cell polarization through phospholipid remodeling. One study shows that SETD2 mutation was associated with the efficacy of immunotherapy, this is due to higher tumor mutation burden 31. It may be used as potential biomarker for cancer immunotherapy in future. SETD2 inactivation in cancer cells can sensitizes cancer cells to immune checkpoint inhibitors. SETD2 was included into a SIGP model to predict immunotherapy outcomes 32.

## 3. EZH2 and Prostate Cancer

### 3.1 Structure and function of EZH2

**Figure 2:**
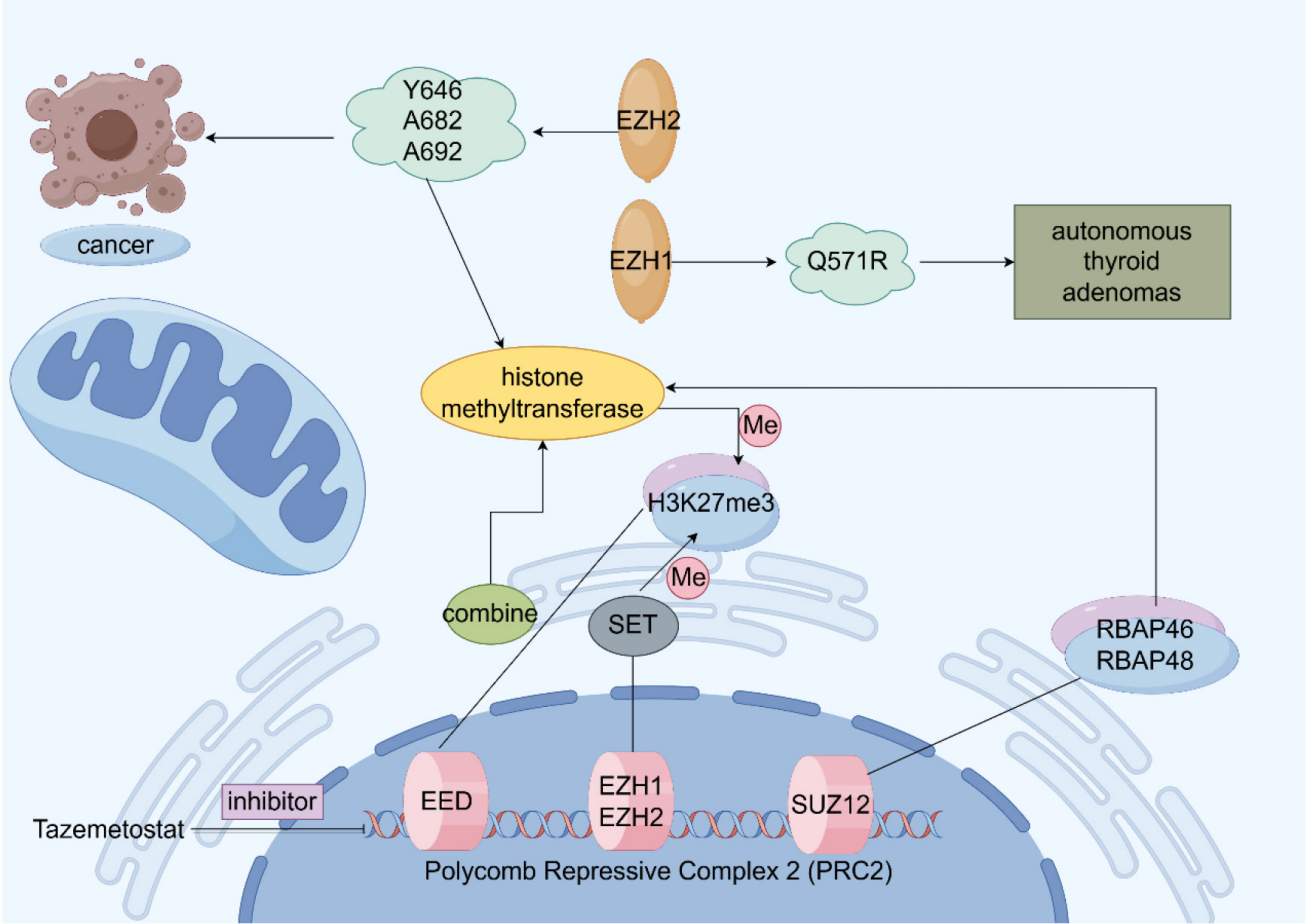
Structure and function of Polycomb Repressive Complex 2 (PRC2). Schematic illustration of the function of enhancer of zeste homolog 2 (EZH2). Activation is indicated by arrows and suppression by blocking lines.

*EZH2* is localized on chromosome 7q35 and contains 20 exons encoding 746 amino acids 33. EZH2 is an important polycomb group (PcG) protein, which is closely associated with epigenetic regulation and epigenetically silences histones by modifying them during transcription. Initially, we discovered PcG proteins in *Drosophila*, where they play a negative regulatory role in gene expression during growth and development 34. In mammals, PcG proteins form two major multiprotein complexes called polycomb repressive complex 1 (PRC1) and PRC2, EZH2 is a key enzyme that catalyzes the trimethylation (H3K27me3) of H3 lysine 27 (H3K27), while EZH2 is the catalytic subunit of PRC2, which mediates histone trimethylation 35-37. H3K27me3 modification can lead to downstream tumor suppressor gene inhibition. The specific trimethylation of H3K27 by EZH2 may be attributed to the fact that EZH2 contains a c-terminal SET structural domain, which ensures that HMTase functions, leading to denser chromatin and epigenetic silencing of the target genes 38-41. In addition to the methyltransferase activity, EZH2 can independently act as a transcription factor involved in epigenetic regulation 42-43. As a major regulator in cell-cycle progression, autophagy, and apoptosis, EZH2 promotes DNA damage repair and inhibits cellular senescence 44-46. Moreover, in gastric cancer cells, EZH2 can bind to the promoter region of tumor suppressor gene *P21* and mediate H3K27me3 modification, resulting in the transcriptional silencing of P21, which, in turn, promotes the proliferation of gastric cancer cells. As *P21* is the key tumor suppressor gene, its function inhibition prevents it from exerting the normal inhibitory effect on tumor cells, which ultimately leads to the abnormal proliferation of gastric cancer cells 47. In addition to histones, EZH2 methylates non-histone proteins in a PRC2-dependent manner (**Figure 2**) 48.

### 3.2 EZH2 and prostate cancer metastasis

Gain-of-function mutations or loss-of-function mutations may occur in EZH2, and both mutation type was involved in the development of prostate cancer. EZH2 functions as an epigenetic writer and AR coregulator, these activities were related to oncogenic activity of EZH2 49. EZH2 usually overexpressed in prostate cancer compared with normal prostate tissue. EZH2 mediate IFN-γ-JAK-STAT1 signaling pathway inactivation to promote prostate cancer progression 50. EZH2 also involves in neuroendocrine prostate cancer progression, this is due to cooperation of EZH2 and N-Myc 51. High expression of EZH2 was also correlated with high risk of recurrence of prostate cancer after radical prostatectomy. EZH2 can activate Ras and NF-κB pathway by epigenetically silencing DAB2IP 52. EZH2 can regulate CDH1 gene expression to affect EMT process of prostate cancer cell. Enzalutamide can induce prostate cancer cell neuroendocrine differentiation via EZH2-STAT3 signaling pathway. One study showed that androgen deprivation can induce neuroendocrine differentiation using CREB-EZH2-TSP1 pathway in prostate cancer 53. Another study demonstrated that T350 phosphorylated EZH2 act with AR to promote lineage plasticity and neuroendocrine differentiation 54. MALAT1, a long-stranded non-coding RNA (lncRNA), is overexpressed in both prostate cancer (PCa) and castration-resistant prostate cancer (CRPC). Studies have demonstrated that MALAT1 binds to EZH2 55 and plays a crucial role in recruiting EZH2 to tumor suppressor gene loci, thereby facilitating PCa cell migration and invasion 55. One of the most prevalent genetic alterations in prostate cancer is the TMPRESS2-ERG fusion, caused by chromosomal translocation on chromosome 21, which has been shown to activate EZH2 expression 56-57. EZH2 is frequently overexpressed in prostate cancer cells and interacts with several DNA methyltransferases (DNMT1, DNMT3A, DNMT3B), reinforcing its role in epigenetic silencing 58. A pivotal cDNA microarray study in 2002 first established the association between EZH2 and prostate cancer, identifying it as the most upregulated gene in metastatic cases 59. Notably, EZH2 overexpression is also detected in localized tumors with a high risk of recurrence after radical prostatectomy 59. EZH2 can act as a coactivator for AR, and promote CRPC progression. Furthermore, EZH2 and BRCA1 cooperate to regulate prostate cancer stem cell phenotype and properties 60.

As a key epigenetic regulator, EZH2 is tightly controlled at transcriptional, translational, and post-translational levels. MicroRNAs (miRNAs) also regulate EZH2 expression post-transcriptionally. For instance, miR-101, miR-26a, and miR-26b —known to suppress EZH2—are significantly downregulated in prostate cancer 61-63. EZH2 can block pro-apoptotic pathway and enhance the anti-apoptotic pathway to induce drug resistance. Loss-of-function mutations in EZH2 promoted resistance to the chemotherapeutic agent Ara C in acute myeloid leukemia. And EZH2 can induce c-Myc overexpression to induce cisplatin resistance in ovarian cancer. And EZH2 is involved in tamoxifen resistance by silencing the expression of GREB1. Inhibition of EZH2 functions can reverse drug resistance to some extent. EZH2 overexpression was one important cause of docetaxel and enzalutamide resistance in prostate cancer 64. Inhibition of EZH2 can enhance the anti-tumor effect of metformin in prostate cancer. EZH2 can regulate the innate and adaptive immune systems of the tumor microenvironment, EZH2 was a driver of resistance to immunotherapies 65. EZH2 overexpressed in tumor cells can inhibit T cell activation by upregulating PD-L1 expression. Otherwise, EZH2 suppression leads to increased activated CD8+ T cells, increased M1 tumor-associated macrophages and enhanced response to PD-1 therapy in prostate cancer. EZH2 also can regulate cytokines and chemokine in the tumor microenvironment 66. EZH2 in cancer cells can regulate NK cell activation in the tumor microenvironment. EZH2 can change the tumor bone microenvironment by regulating the osteoblast and osteoclast when prostate cancer bone metastasis occurs 67. Inhibition of EZH2 can positively activate the immune system toward tumor suppression.

## 4. Inhibitors

### 4.1 Inhibitors of SETD2

SETD2, a lysine N-methyltransferase and the sole enzyme responsible for H3K36 trimethylation, has emerged as a promising therapeutic target due to its role in epigenetic regulation and tumor suppression. It is implicated in various cancers, including multiple myeloma characterized by t (4,14) translocations 68. A selective, potent, and orally bioavailable small-molecule SETD2 inhibitor, EZM0414, has recently shown promise in preclinical studies 68. SETD2 loss is associated with aggressive forms of renal, gastric, colon, and pancreatic cancers 69, and it has been implicated in the development of aggressive gastrointestinal mesenchymal tumors 70. In physiological processes, SETD2 inhibits intestinal epithelial damage by regulating oxidative stress-related factors and plays an inhibitory role in epithelial repair processes 71-72. WEE1 is a class of inhibitory tyrosine kinases, and SETD2 deficiency reduces H3K36me3 levels during cellular physiology, leading to downregulation of the regulatory subunit of ribonucleotide reductase subunit M2 (RRM2) and a subsequent decrease in dNTP availability 73-74. In preclinical experiments, adding a WEE1 kinase inhibitor inhibited the RRM2 expression level, leading to further depletion of the dNTP pool in SETD2-deficient cells (which serves as the single DNA unit), resulting in impaired DNA replication and cell death 75. Recent studies have unveiled that renal cancer cells harboring SETD2 mutations or downregulated SETD2 expression are highly sensitive to histone demethylase inhibitors (HDMIs) in both *in vitro* and *in vivo* models 76-77. Of note, a combination of HDMIs) and poly(adenosine diphosphate-ribose) polymerase inhibitors (PARPi, i.e., HMA + PARPi) exerted synergistic antitumor effects on cell growth by enhancing DNA damage and immune signaling, especially in DNA repair-deficient cancers, such as BRCA-mutated breast cancer 78-79. PARP1 plays a crucial role in homologous recombination (HR)-mediated DNA double-strand break (DSB) repair, which contributes to cancer cell survival in a sustained DNA damage environment. PARPis block PARP1 functions, leading to the accumulation of cytotoxic DSBs and tumor cell death 80-81. Given SETD2's key role in HR and DSB repair, its deficiency may sensitize cancer cells to PARP inhibition, positioning SETD2 as a compelling therapeutic target for such strategies 82-83.

### 4.2 Inhibitors of EZH2

EZH2 overexpression is a hallmark of various cancers and is associated with disrupted methylation patterns that promote tumorigenesis. By silencing tumor suppressor genes, EZH2 enhances cell proliferation, invasion, and progression of cancer cells 84-90. In prostate and breast cancers, EZH2 is phosphorylated by AKT at Ser21, which suppresses its methyltransferase activity by reducing its affinity for histone H3 and leading to diminished H3K27me3 levels. This phosphorylation pEZH2-S21 also results in increased expression of EZH2-repressed genes 91. EZH2 regulates numerous genes that contribute to prostate cancer development by transcriptionally inhibiting tumor metastasis. In prostate cancer, numerous genes have been identified as direct EZH2 targets and are associated with cancer progression and metastasis in the silenced state, which further confirms that EZH2 is a true oncogene. EZH2 can epigenetically silence the dysfunctional homology interaction protein (DAB2IP), a tumor suppressor involved in regulating Ras and NF-κB pathways, thereby driving tumorigenesis and metastasis in prostate cancer 92.

For more than two decades, the compound 3-deazepane-A (DZNep) has been widely recognized as a potent inhibitor of S-adenosine-L-homocysteine (SAH) hydrolase, a cofactor required for EZH2-dependent methylation. Subsequent studies have revealed that DZNep reduces intracellular EZH2 levels and inhibits H3K27me3 93. These findings established DZNep as the first compound to target EZH2. *In vivo* experiments demonstrated that DZNep selectively induces apoptosis in cancer cells without affecting normal cells 93-94. Beyond its anti-proliferative activity, EZH2 inhibition has been shown to suppress migration and invasion of prostate cancer cells 94.

Tazemetostat (Tazverik), is an oral EZH2 inhibitor approved by the U.S. Food and Drug Administration (FDA), for the treatment of follicular lymphoma (FL) and epithelioid sarcoma (ES). In FL, EZH2 activity is enhanced through interactions between EZH2 mutants and wild-type EZH2 (WT EZH2) with oncogenes. Currently, clinical trials are investigating the combination of EZH2 inhibitors with other therapeutic modalities, including immunotherapy, conventional chemotherapy, and targeted therapies 95. Notably, EZH2 inhibitors may exhibit synergistic effects when combined with immunotherapeutic or chemotherapeutic agents.

## 5. Signaling Pathways in Prostate Cancer

EZH2, as the catalytic core subunit of PRC2, is markedly overexpressed in prostate cancer. Moreover, the androgen receptor (AR) is a central therapeutic target in prostate cancer and remains highly expressed even in advanced disease stages 96. AR expression is widespread in both primary and metastatic prostate cancer tissues. Importantly, EZH2 regulates AR protein levels and modulates the transcriptional activity of AR downstream target genes by directly interacting with AR 97. Prostate cancer progression is regulated by the androgens testosterone and 5α-dihydrotestosterone, which exert their effects through binding to AR 98. Ligand binding to AR induces a conformational change in the receptor, releasing auxiliary proteins and promoting AR dimerization. These dimers subsequently translocate to the nucleus and bind to androgen-responsive elements in the promoters of specific genes involved in cell proliferation and apoptosis suppression—key drivers of prostate cancer progression 99-100. The interaction between AR signaling and the tumor microenvironment is highly complex, exhibiting both oncogenic and tumor-suppressive influences 101. Stromal cells play a critical role in prostate development and cancer initiation, mediated in part by AR signaling. Remarkably, AR is undetectable in prostate epithelial tissues during early development, whereas its expression in stromal cells is significantly upregulated 102-103. Consequently, androgen deprivation therapy (ADT) remains a frontline treatment for prostate cancer. Although ADT is initially effective against prostate cancer, resistance inevitably develops, leading to CRPC, which is associated with poor prognosis 104-106.

Recent studies have shown that the PI3K-AKT signaling pathway is significantly aberrantly activated in prostate cancer, particularly in CRPC, and its overactivation is strongly associated with disease progression 107-108. Phosphatidylinositol 3-kinase (PI3K) enzymes, part of the lipid kinase superfamily, are categorized into three classes (I, II, and III) based on their substrate specificity and subunit composition. Their structural differences are predominantly reflected in the combination of catalytic and regulatory subunits 109-110. Class IA PI3K catalyzes the phosphorylation of phosphatidylinositol 4,5-bisphosphate (PIP2) to generate phosphatidylinositol 3,4,5-trisphosphate (PIP3), a key second messenger that activates downstream signaling pathways governing key biological processes such as cell proliferation, autophagy, and apoptosis 111. PIP3 accumulation recruits and activates phosphatidylinositol-dependent kinase 1 (PDK1), which in turn phosphorylates AKT serine/threonine kinase at the Thr308 site. Activated AKT further phosphorylates several downstream effector molecules, such as FOXO transcription factors, glycogen synthase kinase 3β (GSK3β), nuclear factor κB (NF-κB), and tuberous sclerosis complex 2 (TSC2) 112-114. For example, AKT phosphorylates TSC2, inhibiting its GTPase activity and thereby activating Ras homologous enriched enkephalin (RHEB), which in turn prevents the inhibitory effect of RHEB on mTORC1. AKT also suppresses cell autophagy by phosphorylating Unc-51-like autophagy-activating kinase 1 and ribosomal S6 kinase (S6K) and eukaryotic translation initiation factor 4E-binding protein 1 (4EBP1) to synergistically regulate cell growth and ribosome biosynthesis. S6K and 4EBP1 synergistically regulate cell growth, protein translation, and ribosome biosynthesis. Multi-omics analyses have uncovered extensive molecular abnormalities in the PI3K pathway in prostate cancer, including copy number variations, mutations, and transcriptional dysregulation. These abnormalities are present in up to 42% of primary tumor samples and are even more prevalent in metastatic lesions 115-117. The PI3K-AKT-mTOR signaling axis—one of the most frequently deregulated pathways in prostate cancer—contributes to tumor growth and therapy resistance by regulating cell proliferation, survival, and metabolic reprogramming. Despite the development of various targeted agents, including pan-PI3K inhibitors, PI3K subtype-specific inhibitors, AKT inhibitors, mTOR inhibitors, and dual-target inhibitors, their clinical efficacy has been limited. Preclinical studies and early-phase clinical trials have highlighted challenges such as compensatory pathway activation, off-target toxicity, and the complex interplay between tumor cells and the microenvironment 118-120.

## 6. Conclusion

This paper focuses on prostate cancer, examining the epigenetic regulation of SETD2 and EZH2, their roles in metastasis, and the progress of related inhibitors. The discussion provides a multi-dimensional theoretical foundation and practical guidance for the diagnosis, treatment, and development of novel therapeutic strategies for prostate cancer.

SETD2, a critical HMTase, plays an essential and irreplaceable role in epigenetic regulation. In prostate cancer, the SETD2 expression level is significantly reduced and is closely associated with prostate cancer development and metastasis. Its downregulation leads to reduced H3K36me3 levels, disrupting the epigenetic balance and triggering abnormal protein methylation *in vivo*. This dysregulation promotes EMT, enhancing the migratory and invasive capacity of prostate cancer cells and ultimately facilitating metastasis. Additionally, SETD2 loss compromises the fidelity of gene transcription, which, combined with the dysregulation of key oncogenes, further contributes to tumorigenesis. With growing insight into SETD2's mechanisms, it has emerged as a promising therapeutic target. The development of small-molecule SETD2 inhibitors such as EZM0414, along with promising results from combination therapies, offers new avenues for prostate cancer treatment. Combination regimens involving SETD2 inhibitors with WEE1 inhibitors, HDMIs, or PARPis have demonstrated encouraging therapeutic efficacy in preclinical cancer models and are expected to form the basis for new treatment strategies for prostate cancer.

EZH2, the catalytic subunit of PRC2, functions as an oncogene in prostate cancer. It catalyzes H3K27 trimethylation, condensing chromatin and silencing gene expression. It also serves as a transcription regulator involved in diverse biological processes such as cell cycle control, autophagy, and apoptosis. EZH2 is commonly overexpressed in prostate cancer and interacts with various molecular partners. For instance, it binds to the lncRNA MALAT1, promoting PCa cell migration and invasion, and associates with DNA methyltransferases, modulating gene expression. EZH2 expression is tightly regulated at transcription, translation, and post-translational modification levels, with miRNAs playing a notable role in post-transcriptional control. EZH2 upregulation disrupts normal methylation patterns, silences tumor suppressor genes, promotes cell proliferation and invasion, and accelerates cancer progression. The compound DZNep was the first EZH2-targeting agent, known to reduce PRC2 complex protein levels, inhibit H3K27me3, and selectively induce apoptosis in cancer cells without affecting normal cells. Tazemetostat (Tazverik), a U.S. FDA-approved oral EZH2 inhibitor, is already in clinical use for the treatment of specific cancers. Ongoing trials are investigating EZH2 inhibitors in combination with immunotherapy, chemotherapy, and targeted agents. These combination therapies hold promise due to their potential synergistic effects and could offer new hope for patients with advanced disease.

EZH2 also contributes to prostate cancer development by directly interacting with AR, enhancing AR activity, and modulating the transcription of its downstream target genes. While the AR signaling pathway remains a central therapeutic target in prostate cancer, long-term reliance on ADT often leads to CRPC, which is associated with poor clinical outcomes. Concurrently, the PI3K-AKT-mTOR pathway is aberrantly activated in prostate cancer, driving tumor growth and treatment resistance by regulating proliferation, survival, and metabolic reprogramming. Despite the development of numerous inhibitors—targeting PI3K, AKT, mTOR, or combinations thereof—clinical success has been limited. Challenges include compensatory pathway activation, off-target effects, and the complexity of the tumor microenvironment. Consequently, while EZH2, AR, and PI3K-AKT-mTOR represent vital therapeutic targets, their intricate regulatory networks remain significant obstacles to effective treatment.

In summary, SETD2 and EZH2 play distinct but critical roles in prostate cancer development, progression, and metastasis. SETD2 functions as a tumor suppressor, and its loss promotes cancer progression and metastasis. By contrast, EZH2 acts as an oncogene, with its overexpression driving tumor aggressiveness. Deepening our understanding of their molecular mechanisms and advancing targeted therapies against these regulators are crucial for uncovering the pathogenesis of prostate cancer and enabling precision medicine. Future research should focus on exploring the complex regulatory networks of SETD2 and EZH2 in prostate cancer, refining current inhibitor-based treatment regimens, and enhancing combination treatment strategies to improve therapeutic outcomes and patient quality of life.

## 7. Future Directions

The study of epigenetics for carcinogenesis is becoming more and more promising. SETD2 and EZH2 are two important members of HMTs, and plays important roles in prostate cancer progression and metastasis. Mechanistically, SETD2 or EZH2 regulate prostate cancer metastasis and drug resistance warrant further study. And the effect of SETD2 or EZH2 on tumor microenvironment, especially tumor immune microenvironment needs to de deeply studied. Furthermore, tumor cell metabolism is a hot topic in recent years, and whether SETD2 or EZH2 influence tumor cell metabolism and its corresponding mechanism needs to be studied.
